# Active and Passive Exposure to Tobacco and e-Cigarettes During Pregnancy

**DOI:** 10.1007/s10995-020-03037-8

**Published:** 2020-11-19

**Authors:** Laura Schilling, Jacob Spallek, Holger Maul, Marie Tallarek, Sven Schneider

**Affiliations:** 1 Department of Public Health, Brandenburg University of Technology, Universitätsplatz 1, 01968 Senftenberg, Germany; 2 Department of Public Health, Brandenburg University of Technology, Senftenberg, Germany; 3 Asklepios Klinik Barmbek, Hamburg, Germany; 4 Mannheim Institute of Public Health, Social and Preventive Medicine, Medical Faculty Mannheim, Heidelberg University, Mannheim, Germany

**Keywords:** E-cigarette, Tobacco cigarette, Nicotine, Passive exposure, Pregnancy

## Abstract

**Objectives:**

Active and passive exposure to tobacco cigarettes during pregnancy is associated with multiple negative health outcomes for the fetus. In addition, exposure to e-cigarettes has been progressively discussed as a new threat to fetal health. Until now, there has been a lack of studies examining active and passive exposure to tobacco and e-cigarettes among pregnant women. The objective of our current **ST**udy on **E**-cigarettes and **P**regnancy (STEP) was to advance and complement the current knowledge regarding active and passive exposure to tobacco and e-cigarettes before pregnancy and during early and late pregnancy.

**Methods:**

One element of the STEP study was a quantitative cross-sectional design: A sample of 540 pregnant women recruited at an obstetrician clinic in Hamburg from April 2018 to January 2019 were surveyed once via a standardized questionnaire and provided complete information regarding their consumption of tobacco and e-cigarettes. We performed a descriptive analysis of tobacco and e-cigarette use before pregnancy and during early and late pregnancy, as well as bivariate analysis of these variables with sociodemographic determinants. Passive exposure was assessed by asking the participating pregnant women about the consumption of tobacco and e-cigarettes by their partners, in general, and in their homes.

**Results:**

Before pregnancy, 20.0% of the participants used tobacco cigarettes exclusively, 1.3% used e-cigarettes exclusively, and 6.5% were dual users. Educational level was significantly associated with tobacco cigarette use (p < 0.001) and dual use (p = 0.047) before pregnancy. During early (late) pregnancy, 8.7% (2.8%) used tobacco cigarettes and 0.4% (0.0%) used e-cigarettes exclusively. Twenty-point nine percent of women’s partners consumed tobacco cigarettes exclusively, 2.7% consumed e-cigarettes exclusively, and 2.7% consumed both. A total of 8.5% (16.7%) of the partners who consumed tobacco cigarettes exclusively (e-cigarettes exclusively) did so in the women’s homes.

**Conclusions for Practice:**

Among pregnant women, the use of tobacco cigarettes remains prominent before and during pregnancy, while e-cigarette use predominately occurs before pregnancy. Our study shows that pregnant women are frequently exposed to their partners’ tobacco and e-cigarette use within their homes. Strategies to reduce such exposure should be further intensified.

## Significance

In the last decade, e-cigarettes have become popular, and e-cigarette use has been increasing, even among pregnant women. However, there is a lack of knowledge concerning the exposure to e-cigarettes before pregnancy and during early and late pregnancy, as well as studies comparing e-cigarettes and tobacco cigarettes. The current study found that active exposure to e-cigarettes before pregnancy is high, while exposure during pregnancy is marginal. In contrast, exposure to tobacco cigarettes before and in early pregnancy is high but decreases during late pregnancy. In addition, the results of our study show that pregnant women are passively exposed to tobacco and e-cigarettes at home. Pregnant women should be advised against exposing themselves, actively or passively, to tobacco and e-cigarette smoke.

## Introduction

Active and passive tobacco smoking during pregnancy is a significant health risk for pregnant women and their fetuses (DKFZ 2003; Leonardi-Bee et al. 2011; U.S. Department of Health and Human Services 2014). Maternal tobacco smoking can lead to premature birth, spontaneous abortion, malformations, cognitive delays, and sudden infant death (Andres and Day 2000; Habek et al. 2002; Kyrklund-Blomberg et al. 2001). In addition, passive exposure to tobacco cigarettes during pregnancy is an inherent threat to maternal and fetal health (Leonardi-Bee et al. 2011). However, pregnant women remain actively and passively exposed to tobacco smoke. According to representative data from 2007 to 2016, nearly 11% of pregnant women in Germany smoked tobacco cigarettes (Kuntz et al. 2018). Moreover, 2% to 6% of nonsmoking women are passively exposed to tobacco smoke at home for at least one hour per week (Zeiher et al. 2018). Hence, both active and passive exposure to tobacco smoke during pregnancy should be strongly monitored and eliminated.

As a relatively new product, electronic cigarettes (e-cigarettes) should be considered in such monitoring. E-cigarette exposure during pregnancy is a risk factor for both maternal and fetal health. Early research studies with animals showed that in utero exposure to e-cigarettes can lead to various negative health outcomes including epigenetic, organic, and pulmonary outcomes (Chen et al. 2018; Hess et al. 2016; McGrath-Morrow et al. 2015). Concurrently, international studies estimated the prevalence of active e-cigarette use during pregnancy to be between 0.5% and 15% (Bhandari et al. 2018; Kapaya et al. 2019; Mark et al. 2015; Wagner et al. 2017). Moreover, previous studies have indicated that e-cigarettes are used as a means to quit smoking by women during pregnancy (Bowker et al. 2018; McCubbin et al. 2017) and have discussed smaller perceived health risks, as compared to tobacco cigarettes as a reason for e-cigarette use among pregnant women (Bowker et al. 2018; McCubbin et al. 2017).

In order to monitor and eliminate tobacco and e-cigarette exposure during pregnancy as early as possible, it is crucial to gain detailed knowledge about women’s consumption patterns before and during the course of pregnancy (e.g., before pregnancy, during the first trimester, second trimester, and third trimester) and in relation to sociodemographic determinants. However, previous studies do not provide a complete picture of these aspects. Kapaya et al. (2019), for example, examined consumption patterns of tobacco and e-cigarettes three months before pregnancy and during the last three months of pregnancy among a collective of pregnant women from Texas and Oklahoma. Three other research studies (Bhandari et al. 2018; Kurti et al. 2018, 2017) examined the prevalence of e-cigarette use among pregnant women from the U.S. who were current, former, or never previously users of tobacco cigarettes. Unfortunately, these studies did not examine potential sociodemographic predictors of consumption behavior, even though numerous studies have shown that tobacco cigarette use during pregnancy varies according to age, educational level, migration background and marital status/partnership status, among others (Kuntz et al. 2018; Murin et al. 2011). Furthermore, an important predictor is pregnant women’s partners’ consumption behavior. Pregnant women who smoke tobacco cigarettes often have partners who also actively smoke tobacco cigarettes (Schneider and Schütz 2008). In addition, partners’ consumption of tobacco cigarettes has been shown to be the most common source of passive exposure to tobacco smoke during pregnancy (Eiden et al. 2011).

To our knowledge, no studies have examined active and passive tobacco and e-cigarette exposure within one sample of pregnant women. Studies examining changes in tobacco and e-cigarette use from before pregnancy to early and late pregnancy are also lacking. Therefore, our first aim was to examine consumption patterns of tobacco and e-cigarettes at three points in time (the year before, during the first trimester, and during the remainder of pregnancy) and to describe changes in these behaviors over time. Our second aim was to determine the consumption patterns of tobacco and e-cigarettes among partners of pregnant women, in general, and in the women’s homes. Our third aim was to identify if factors such as age, immigrant background, educational level, partnership status, and consumption behavior of the partner are associated with consumption patterns of tobacco and e-cigarettes before pregnancy and during early and late pregnancy.

## Methods

### Data Collection

Our analyses were based on primary data of our currently conducted quantitative survey of pregnant women, which is part of our sequential Mixed-Method-Study STEP (“Study on E-Cigarettes and Pregnancy”). The aims of STEP are to extensively explore risk perceptions and health beliefs regarding e-cigarette use during pregnancy based on an Integrated Health Belief Model (IHBM) and to examine consumption patterns of tobacco and e-cigarettes. A previously published study protocol describes the study design of the STEP in detail (Schilling et al. 2020).

The STEP participants for the cross-sectional quantitative study part were recruited from the Asklepios Klinik Barmbek in Hamburg, Germany. All women who registered for birth at the clinic between April 4th, 2018, and January 11th, 2019, were eligible to participate in the survey. Further selection criteria included an age > 17 years, the ability to complete a questionnaire in German, and providing informed consent prior to their inclusion in the study.

The STEP participants were surveyed once via a standardized questionnaire, for example, about their use of e-cigarettes before pregnancy and during their early and late pregnancy. The questionnaire was based on an extensive literature review, a previously conducted qualitative study and on items and instruments that were field-tested in other studies. In addition, the questionnaire underwent expert review and was pretested with 10 pregnant women outside the study sample of the Asklepios Klinik Barmbek. The Medical Ethics Committee of the Medical Faculty in Mannheim, Heidelberg University (2017-505 N-MA) provided an initial study approval, which was reviewed and approved by the Ethics Committee of the Hamburg Medical Chamber (MC-178/17). The study was performed in accordance with the ethical standards laid down in the Declaration of Helsinki (1964) and its later amendments.

A total of 2092 pregnant women registered for birth during the above-mentioned study period, of which 575 responded to the questionnaire (response rate: 27.5%). The following analysis was based on 540 participants, who provided full information on their consumption behavior of tobacco and e-cigarettes. For more details about the response rate, see the study protocol (Schilling et al. 2020).

### Measurement and Operationalization

#### Active Tobacco and e-Cigarette Use

The use of tobacco cigarettes before pregnancy and during early and late pregnancy was assessed using three items: “Did you smoke tobacco cigarettes at any time in the year before pregnancy?,” “Did you smoke tobacco cigarettes during the first three months of pregnancy?,” and “Did you smoke tobacco cigarettes during the remainder of pregnancy”? (SPATZ Studie 2018). The response categories were “Yes” and “No.” In addition, we asked for the number of cigarettes smoked per day before pregnancy and during early and late pregnancy, with the item referring to each time period: “If yes, please indicate how many tobacco cigarettes you smoked.” (SPATZ Studie 2018). The responses to these items were “up to 5,” “between 6 and 10,” “between 11 and 20,” and “more than 20.”

E-cigarette use before pregnancy and in early and late pregnancy was measured similarly to tobacco cigarette use with the items: “Did you use e-cigarettes at any time in the year before pregnancy?,” “Did you use e-cigarettes during the first three months of pregnancy?,” “Did you use e-cigarettes in the remainder of pregnancy”? Response categories were “Yes, occasionally,” “Yes, regularly,” and “No.”

#### Active Tobacco and e-Cigarette Use of the Partners, in General, and in the Study Participants’ Homes

The partners’ use of tobacco cigarettes was assessed by the following questions: “Does your partner currently smoke tobacco cigarettes”? (Spallek et al. 2017). Response categories were “Yes” and “No.” This item was followed by the item: “If yes, does your partner smoke in your flat/house”? E-cigarette use of the partner, in general, and in the women’s flat/house was assessed by similar items referencing e-cigarettes.

### Sociodemographic Characteristics

We assessed several sociodemographic characteristics, including age, immigrant background and educational level. Immigrant background was assessed according to the commonly used basic set of indicators for mapping migrant status developed by Schenk and Bau (2006). Educational level was categorized as low (still at school, without a school-leaving qualification, or general school graduation [Hauptschulabschluss]), medium (secondary school graduation [Realschulabschluss]), and high (high school graduation [Abitur]). In addition, we asked the participants about their marital status and formed a binary indicator for having a partner based on marital status. The participants were considered to have partners (these could be male/female) if they responded that they were “married” or “non-married, in a partnership.” Furthermore, we assessed the week of pregnancy and the number of pregnancies for the sample description.

#### Data Analysis

First, we performed a descriptive analysis of the consumption behavior of tobacco and e-cigarettes and the number of smoked tobacco cigarettes before pregnancy and during early and late pregnancy. In relation to this descriptive analysis, we analyzed the change in tobacco and e-cigarette consumption from before pregnancy to late pregnancy, including the percentage of quitters and the percentage of individuals who increased or decreased their amount of smoked tobacco cigarettes per day. Second, we descriptively analyzed the partners’ tobacco and e-cigarette consumption, in general, and in the women’s homes. Third, we analyzed the identified consumption patterns (exclusive tobacco cigarette use, exclusive e-cigarette use, and dual use) before pregnancy and during early and late pregnancy by sociodemographic characteristics and the partners’ consumption behavior by using a two-sided chi-square test/Fisher’s exact test.

## Results

### Sample Characteristics

The mean age of the surveyed pregnant women was 32.27 (SD 4.68) years, and the mean pregnancy week was 32.29 (2.75) weeks. In total, 96.1% of the participants had a partner, and 26.2% were classified as having an immigrant background. More than two-thirds (68.9%) had a high level of education, and a fifth (25.6%) had a medium level of education. Most participants had no children (58.1%).

### Consumption Behavior Before and During Pregnancy

**Fig. 1:**
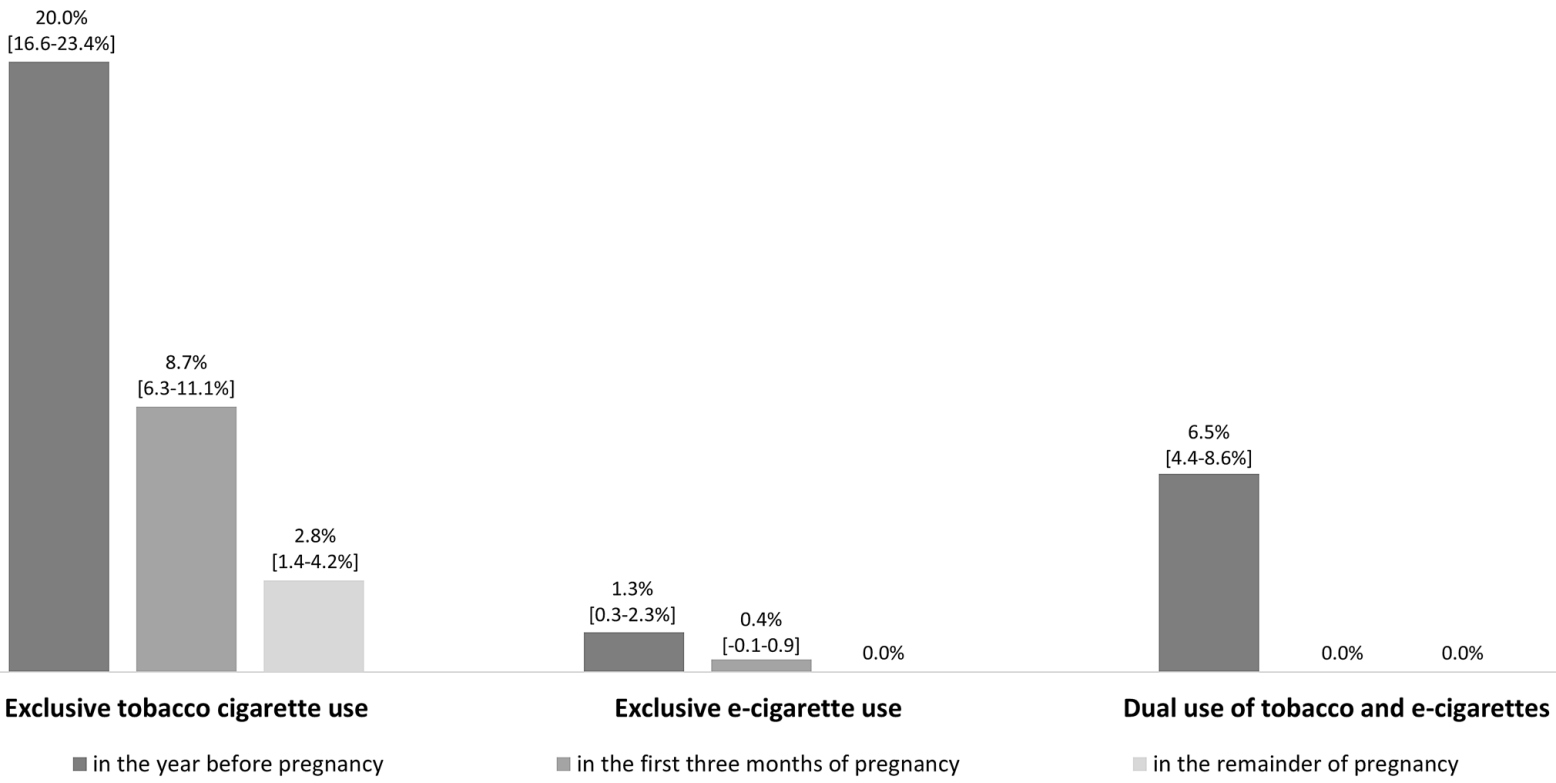
Consumption behavior before pregnancy, during early and late pregnancy (n = 540)

**Fig. 2:**
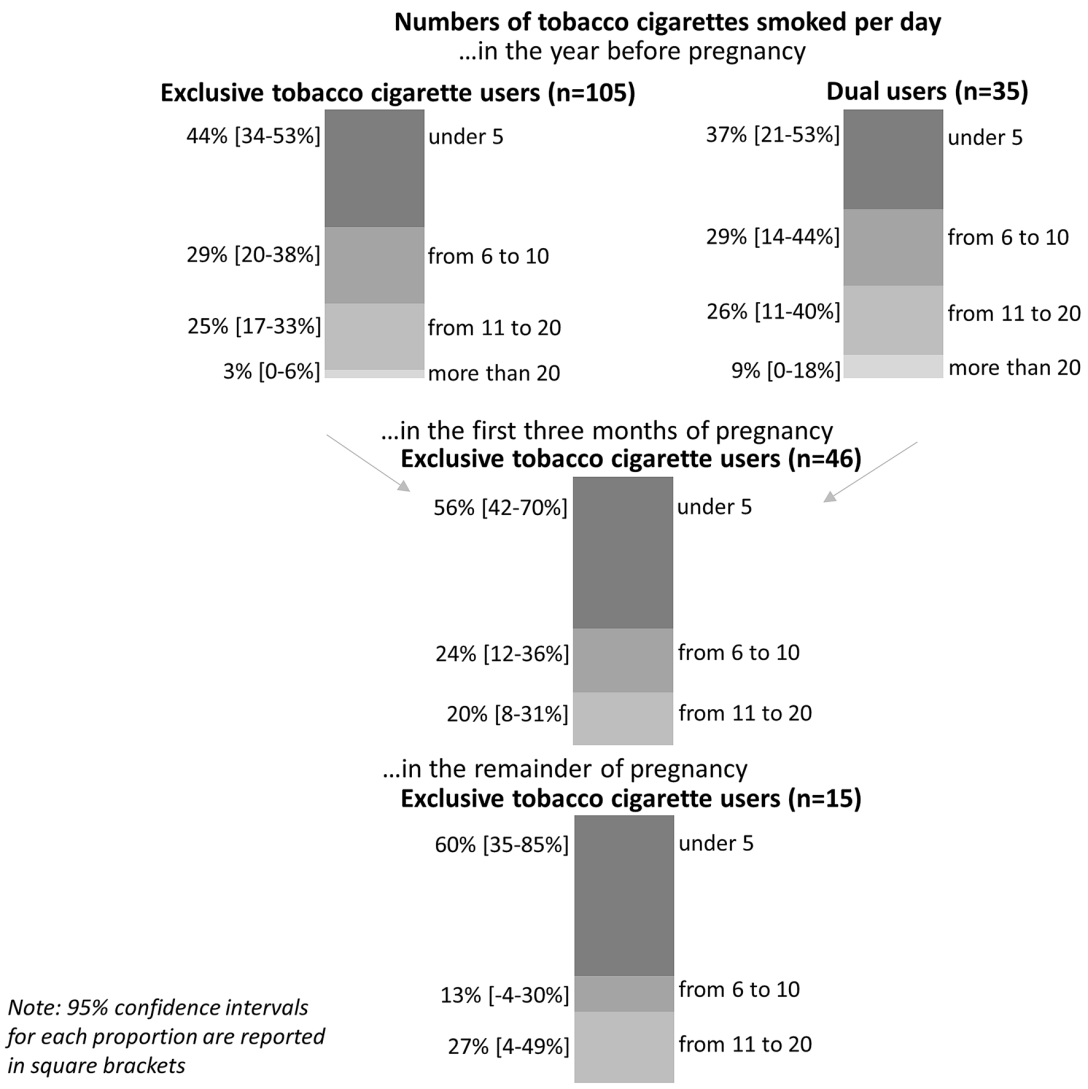
Number of tobacco cigarettes smoked per day before pregnancy, during early and late pregnancy

A large number of the participants were exclusive tobacco cigarette users (20.0%) or dual users (6.5%) before pregnancy, while exclusive e-cigarette users (1.3%) before pregnancy were less common (Fig. 1). The number of individuals who consumed more than ten tobacco cigarettes per day was high (exclusive tobacco cigarette users: 28%; dual user: 35%) (Fig. 2).

The prevalence of exclusive tobacco cigarettes, exclusive e-cigarettes, and dual use declined in the first trimester. Ninety-seven-point one percent of dual users quit e-cigarettes in the year before pregnancy, and 57.1% quit smoking cigarettes before pregnancy. Among those who exclusively used tobacco cigarettes before pregnancy, 70.4% quit smoking, and no women switched to e-cigarettes. In summary, the prevalence of exclusive tobacco cigarette use was high (8.7%) in the first three months of pregnancy, while exclusive e-cigarette use was rare (0.4%), and no participants were dual users (0.0%) (Fig. 1). Almost half (48.4%) of those who exclusively used tobacco cigarettes in the first three months of pregnancy reduced their number of tobacco cigarettes, and 20% continued to smoke more than 10 cigarettes per day (Fig. 2).

In total, in the first three months, 31.9% of exclusive tobacco cigarette users did not stop using tobacco cigarettes for the remainder of their pregnancies. Accordingly, the prevalence of exclusive tobacco cigarette use in the remainder of pregnancy was 2.8% (Fig. 1). We identified that 80% of these participants did not increase or decrease their number of smoked cigarettes per day during the remainder of their pregnancy. In total, during the remainder of their pregnancy, 27% of tobacco cigarette users consumed more than 10 cigarettes per day (Fig. 2).

### Partners’ Consumption Behavior, in General, and in the Participants’ Homes

The prevalence of exclusive tobacco cigarette use was relatively high (20.9%) among the participants’ partners, while the use of e-cigarettes (2.7%) or dual use was less common (2.7%). More than one in ten of the consuming partners used tobacco or e-cigarettes in the women’s homes (Table 1).

**Table 1:** Consumption patterns of the partners, in general, and in the women’s homes (n = 517)

	Exclusive tobacco cigarette use	Exclusive e-cigarette use	Dual use of tobacco and e-cigarettes
	n (%)	n (%)	n (%)
Use, in general	107 (20.9)	14 (2.7)	14 (2.7)
of these: Use in the women’s homes	9 (8.5)	2 (16.7)	1 (7.1) (Tob-cig) 3 (21.4) (E-cig)

Percentages are based on valid cases

### Factors Determining Consumption Behavior Before and During Pregnancy

The participants’ educational levels were significantly associated with their consumption behavior before and during pregnancy. For example, participants with a low or medium educational level (10.7%) were more likely to be dual users before pregnancy compared to those participants with a high educational level (4.8%) (p = 0.047) (Table 2). Participants with a low educational level (28.6%) were more likely to consume tobacco cigarettes during early pregnancy compared to individuals with a medium (17.6%) or high educational level (4.0%) (p < 0.001), while not having a partner (p = 0.017) was associated with tobacco cigarette use during late pregnancy (Table 1). Pregnant women with a partner who used tobacco cigarettes exclusively were more likely to exclusively consume tobacco cigarettes before pregnancy (p < 0.001) and during early (p < 0.001), and late pregnancy (p < 0.001).

**Table 2:** Consumption patterns of the pregnant women by sociodemographic characteristics and consumption behavior of the partners

		Consumption patterns before pregnancy						Consumption patterns during early pregnancy				Consumption patterns during late pregnancy	
Sociodemographic characteristics		Exclusive tobacco cigarette use		Exclusive e-cigarette use		Dual use		Exclusive tobacco cigarette use		Exclusive e-cigarette use		Exclusive tobacco cigarette use	
		n (%)	p (χ^2^)	n (%)	^1^	n (%)	p (χ^2^)	n (%)	p (χ^2^)	n (%)	^1^	n(%)	p (χ^2^)
Total	540 (%)	108 (20.0)		7 (1.3)		35 (6.5)		47 (8.7)		2 (0.4)		15 (2.8)	
Age group (in years)			**.013**		/		.218		.286		/		.144^3^
18 to 29	156 (29.1)	43 (27.6)		4 (2.6)		14 (9.0)		17 (10.9)		1 (0.6)		7 (4.5)	
30 to 34	204 (38.0)	39 (19.1)		2 (1.0)		9 (4.4)		13 (6.4)		0 (0.0)		2 (1.0)	
> 34	177 (33.0)	26 (14.7)		1 (0.6)		12 (6.8)		17 (9.6)		1 (0.6)		6 (3.4)	
Immigrant background			.271		/		.695		.385		/		**.031**^**3**^
No	398 (73.8)	75 (18.8)		3 (0.8)		25 (6.3)		32 (8.0)		1 (0.3)		7 (1.8)	
Yes	141 (26.2)	33 (23.4)		4 (2.8)		10 (7.1)		15 (10.6)		1 (0.7)		8 (5.7)	
Educational level			** < .001**		/		**.047**		** < .001**		/		** < .001**^**3**^
Low	28 (5.5)	13 (46.4)		3 (10.7)		3 (10.7)		8 (28.6)		1 (3.6)		7 (25.0)	
Medium	131 (25.6)	37 (28.2)		1 (0.8)		14 (10.7)		23 (17.6)		0 (0.0)		5 (3.8)	
High	353 (68.9)	53 (15.0)		2 (0.6)		17 (4.8)		14 (4.0)		1 (0.3)		3 (0.8)	
Having a partner			.589^3^		/		.150^3^		.101^3^		/		**.017**^**3**^
Yes	517 (96.1)	103 (19.9)		6 (1.2)		32 (6.2)		43 (8.3)		2 (0.4)		12 (2.3)	
No	21 (3.9)	5 (23.8)		1 (4.8)		3 (14.3)		4 (19.0)		0 (0.0)		3 (14.3)	
Exclusive tobacco cigarette use of the partners^2^			** < .001**		/		.509		** < .001**		/		**.001**^**3**^
No	404 (79.1)	60 (14.9)		5 (1.2)		24 (5.9)		23 (5.7)		0 (0.0)		4 (1.0)	
Yes	107 (20.9)	43 (40.2)		1 (0.9)		8 (7.5)		20 (18.7)		2 (1.9)		8 (7.5)	
Exclusive e-cigarette use of the partners^2^			1.000^**3**^		/		.600^3^		1.000^3^		/		**1.000**^**3**^
No	497 (97.3)	100 (20.1)		6 (1.2)		31 (6.2)		42 (8.5)		2 (0.4)		12 (2.4)	
Yes	14 (2.7)	3 (21.4)		0 (0.0)		1 (7.1)		1 (7.1)		0 (0.0)		0 (0.0)	
Dual use of the partners^2^			**.043**^**3**^		/		.051^3^		**.023**^**3**^		/		1.000^3^
No	497 (97.3)	97 (19.5)		5 (1.0)		29 (5.8)		39 (7.8)		2 (0.4)		12 (2.4)	
Yes	14 (2.7)	6 (42.9)		1 (7.1)		3 (21.4)		4 (28.6)		0 (0.0)		0 (0.0)	

Percentages are based on valid cases

^1^Due to the small number of cases we did not calculate a Pearson Chi-square test/Fisher’s exact test

^2^Participants without a partner were excluded (n = 517)

^3^ ≥ 25% of expected frequencies less than 5

## Discussion

The aim of our study was to determine the patterns of tobacco and e-cigarette consumption the year before pregnancy and during early and late pregnancy. Our analysis revealed that the exclusive use of tobacco cigarettes before and during early pregnancy was a prominent pattern among the surveyed pregnant women, while dual use was limited to the time prior to pregnancy. In addition, our study identified a steep decrease in exclusive tobacco cigarette use from early to late pregnancy. However, tobacco cigarette users in late pregnancy consumed a large amount of tobacco cigarettes per day.

Our results indicate that the consumption of tobacco cigarettes during pregnancy remains a problematic pattern in Germany, while the consumption of e-cigarettes (exclusively or dual) during pregnancy is marginal. With the exception of a study conducted by Wagner et al. (2017), who identified dual use as a prominent pattern during pregnancy—existing studies examining patterns of tobacco and e-cigarette use are in line with our results (Kapaya et al. 2019; Kurti et al. 2018). However, our findings contradict previous indications that pregnant women might use e-cigarettes as a means to quit smoking during pregnancy (England et al. 2016; Mark et al. 2015; McCubbin et al. 2017). Based on our results, only one woman switched from dual use before pregnancy to e-cigarettes exclusively during pregnancy. Hence, strategies to reduce tobacco cigarette use during pregnancy should be further intensified. Nevertheless, e-cigarette exposure during pregnancy should also be further monitored and pregnant women should be advised against using e-cigarettes during pregnancy.

We observed an association between tobacco cigarette use before pregnancy and during pregnancy with age, which was consistent with previous studies (Schneider and Schütz 2008). However, regarding dual use, a different picture emerged, as dual use before pregnancy was not significantly associated with age. Nevertheless, we could identify tendencies that showed that younger women were more commonly dual users before pregnancy. This finding supports a previous study by Ashford et al. (2017), who identified a younger age as a predictor of e-cigarette use in a U.S. collective of women of childbearing age. In addition, educational level was an important predictor of dual use before pregnancy and tobacco cigarette use before and during pregnancy. While research on the predictors of dual use before or during pregnancy is scarce, our results are in line with previous studies on tobacco cigarette use (Graham et al. 2010; Kharkova et al. 2016). Moreover, we identified that having a smoking partner (exclusively or dual) was associated with smoking before and during pregnancy, which was consistent with previous studies (Murin et al. 2011).

The prevalence of tobacco cigarette use (exclusive or dual) among the participants’ partners was somewhat lower compared to former national figures (our study: 23.6%; previous studies: 27.0%–32.2%) (Kotz et al. 2018; Zeiher et al. 2017). At the same time, the estimates for e-cigarette use (single or dual) in our studies were higher (our study: 5.5%; DEBRA: male 2.6%, DKFZ: 1.2% [male and female]) (DKFZ 2018; Kotz et al. 2018). These differences in prevalence may result from two aspects. First, previous studies showed that partners of pregnant women are willing to make behavioral changes and partially quit smoking during pregnancy (Everett et al. 2005). Second, previous studies have also indicated that e-cigarettes would be less harmful and could help users to quit smoking tobacco cigarettes (Baeza-Loya et al. 2014; Farsalinos et al. 2016). Therefore, partners of pregnant women might switch to e-cigarettes in order to quit tobacco cigarette use during their partner’s pregnancy. Further research should explore in greater detail if and why partners of pregnant women use e-cigarettes.

Our study showed that the participants were partially exposed to tobacco smoke by their partners. Comparing our results with previous studies (Rebhan et al. 2009) it appears that the passive smoking of pregnant women at home has declined. These results seem to be in line with studies on the general population in Germany, which showed a decline in passive tobacco exposure at home from 2007 to 2014 (Zeiher et al. 2018). The lower exposure observed in our study may result from intensive legal efforts and educational attempts based on the protection of nonsmokers since 2007 (Zeiher et al. 2018). Despite these efforts, e-cigarettes form a new source of passive exposure that needs to be considered. We found that every fifth pregnant woman with a partner consuming e-cigarettes is passively exposed to their smoking. Previous studies have shown that passive exposure to e-cigarettes can be harmful to humans (Hess et al. 2016). Consequently, future educational and preventive measures should work towards reducing the use of tobacco and e-cigarettes in pregnant women’s environments.

To our knowledge, this study is the first to explore active and passive consumption patterns of tobacco and e-cigarettes within one sample of pregnant women. However, our results should be interpreted with several limitations in mind. First, the representativeness of the findings is limited as our sample was limited to one hospital in Germany. In addition, the proportion of participants with a low educational level in our sample was 3% points lower than the proportion found in the background population of women aged 20 to 39 years in Hamburg (Statistisches Amt für Hamburg und Schleswig–Holstein 2019), and 12% points lower compared to national figures on women between 20 and 40 years of age (Statistisches Bundesamt 2018). Since our results showed that pregnant women with a low educational level smoke more frequently, our observed prevalence may be underestimated. However, our identified prevalence estimates of tobacco cigarette use were in line with studies reporting actual prevalence estimates of tobacco cigarette use before (27.0%) and during pregnancy in Germany (7.5% to 10.9%) (Braig et al. 2019; Grosser 2016; Kuntz et al. 2018). In addition, the low participation rates of women with a low educational level in their respective research studies is a known problem in Germany (Braig et al. 2019; Spallek et al. 2017). Second, the prevalence of substance use in our study could be underestimated, as the results are based on self-reported data. Social desirability may have affected the responses of the surveyed participants. While it was not possible to perform a bio-chemical validation of tobacco and e-cigarette user status, we carried out other strategies to reduce this bias. We informed the participants about the study’s independence from the clinical environment, which was guaranteed by involving an independent research team that opened and analyzed the enveloped survey packages outside the clinic. Third, recall bias could have under- or overestimated the observed prevalence rates. Participants may have faced difficulties recalling their smoking behavior before or at the beginning of their pregnancy. However, since our questions referred to well-recognizable or current behaviors, this limitation seems to be negligible. Fourth, the validity of our bivariate results is limited since the expected frequencies within the chi-square test were partially less than five. If possible, we performed Fisher’s exact test to eliminate this limitation. Fifth, our results are partially limited, as we did not perform multivariate analyses to validate the associations observed in the bivariate analyses due to small cell sizes. Sixth, we limited our analysis to cross-sectional data only providing the first status quo of behaviors, which may change significantly over time. For this reason, our cross-sectional study does not support causal inferences.

In conclusion, our results show that tobacco cigarette use during pregnancy remains an important problematic consumption behavior during pregnancy. In contrast, the use of e-cigarettes was marginal in our sample. With regard to passive exposure, our results show a number of pregnant women are exposed to tobacco and e-cigarettes at home. Therefore, strategies to reduce tobacco cigarette use during pregnancy should be further intensified. Educational and preventive measures should advise against the use of tobacco and e-cigarettes in pregnant women’s environments.

## References

[CR1] Andres RL, Day MC (2000). Perinatal complications associated with maternal tobacco use. Seminars in Neonatology.

[CR2] Ashford K, Rayens E, Wiggins AT, Rayens MK, Fallin A, Sayre MM (2017). Advertising exposure and use of e-cigarettes among female current and former tobacco users of childbearing age. Public Health Nursing.

[CR3] Baeza-Loya S, Viswanath H, Carter A, Molfese DL, Velasquez KM, Baldwin PR, Thompson-Lake DGY (2014). Perceptions about e-cigarette safety may lead to e-smoking during pregnancy. Bulletin of the Menninger Clinic.

[CR4] Bhandari NR, Day KD, Payakachat N, Franks AM, McCain KR, Ragland D (2018). Use and risk perception of electronic nicotine delivery systems and tobacco in pregnancy. Womens Health Issues.

[CR5] Bowker K, Orton S, Cooper S, Naughton F, Whitemore R, Lewis S, Bauld L (2018). Views on and experiences of electronic cigarettes: A qualitative study of women who are pregnant or have recently given birth. BMC Pregnancy and Childbirth.

[CR6] Braig S, Stalder T, Kirschbaum C, Rothenbacher D, Genuneit J (2019). The association of potential stressors with hair steroids in parents with small children: The Ulm SPATZ health study. Psychoneuroendocrinology.

[CR7] Chen H, Li G, Chan YL, Chapman DG, Sukjamnong S, Nguyen T, Annissa T (2018). Maternal e-cigarette exposure in mice alters DNA methylation and lung cytokine expression in offspring. American Journal of Respiratory Cell and Molecular Biology.

[CR8] DKFZ (2003). Passivrauchende Kinder in Deutschland - frühe Schädigungen für ein ganzes Leben [Passive smoking children in Germany - early damage for the whole life].

[CR9] DKFZ. (2018). *E-Zigaretten: Konsumverhalten in Deutschland 2014–2018. Aus der Wissenschaft - für die Politik. [E-cigarettes: Consumption behavior in Germany 2014–2018. From sciences - for politics]*. Heidelberg: Deutsches Krebsforschungszentrum.

[CR10] Eiden RD, Molnar DS, Leonard KE, Colder CR, Homish GG, Maiorana N, Schuetze P (2011). Sources and frequency of secondhand smoke exposure during pregnancy. Nicotine and Tobacco Research.

[CR11] England LJ, Tong VT, Koblitz A, Kish-Doto J, Lynch MM, Southwell BG (2016). Perceptions of emerging tobacco products and nicotine replacement therapy among pregnant women and women planning a pregnancy. Preventive Medicine Reports.

[CR12] Everett KD, Gage J, Bullock L, Longo DR, Geden E, Madsen RW (2005). A pilot study of smoking and associated behaviors of low-income expectant fathers. Nicotine and Tobacco Research.

[CR13] Farsalinos KE, Poulas K, Voudris V, Le Houezec J (2016). Electronic cigarette use in the European Union: Analysis of a representative sample of 27460 Europeans from 28 countries. Addiction.

[CR14] Graham H, Hawkins SS, Law C (2010). Lifecourse influences on women's smoking before, during and after pregnancy. Social Science & Medicine.

[CR15] Grosser, A. (2016). *Determinanten von subjektivem Stress in der Schwangerschaft. Eine Auswertung von Baseline-Daten der sozialepidemiologischen Geburtskohorte zur "Gesundheit von Babys und Kindern in Bielefeld" (BaBi-Studie). [Determinants of subjective stress during pregnancy. A analysis of the baseline data of the social-epidemiological birth cohort "Health of Babies in Bielefeld" (The BaBi-Study)]*. Bielefeld: Universität Bielefeld.

[CR16] Habek D, Habek JC, Ivanisevic M, Djelmis J (2002). Fetal tobacco syndrome and perinatal outcome. Fetal Diagnosis and Therapy.

[CR17] Hess IM, Lachireddy K, Capon A (2016). A systematic review of the health risks from passive exposure to electronic cigarette vapour. Public Health Research and Practice.

[CR18] Kapaya M, D'Angelo DV, Tong VT, England L, Ruffo N, Cox S, Warner L (2019). Use of electronic vapor products before, during, and after pregnancy among women with a recent live birth—Oklahoma and Texas, 2015. Mmwr-Morbidity and Mortality Weekly Report.

[CR19] Kharkova OA, Krettek A, Grjibovski AM, Nieboer E, Odland JO (2016). Prevalence of smoking before and during pregnancy and changes in this habit during pregnancy in Northwest Russia: a Murmansk county birth registry study. Reproductive Health.

[CR20] Kotz D, Böckmann M, Kastaun S (2018). The use of tobacco, e-cigarettes, and methods to quit smoking in Germany. Deutsches Ärzteblatt International.

[CR21] Kuntz B, Zeiher J, Starker A, Prütz F, Lampert T (2018). Rauchen in der Schwangerschaft - Querschnittsergebnisse aus KIGGS Welle 2 und Trends [Smoking during pregnancy - Cross-sectional results of KIGGS wave 2 and trends]. Journal of Health Monitoring.

[CR22] Kurti AN, Bunn JY, Villanti AC, Stanton CA, Redner R, Lopez AA, Gaalema DE (2018). Patterns of single and multiple tobacco product use among US women of reproductive age. Nicotine and Tobacco Research.

[CR23] Kurti AN, Redner R, Lopez AA, Keith DR, Villanti AC, Stanton CA, Gaalema DE (2017). Tobacco and nicotine delivery product use in a national sample of pregnant women. Preventive Medicine.

[CR24] Kyrklund-Blomberg NB, Gennser G, Cnattingius S (2001). Placental abruption and perinatal death. Paediatric and Perinatal Epidemiology.

[CR25] Leonardi-Bee J, Britton J, Venn A (2011). Secondhand smoke and adverse fetal outcomes in nonsmoking pregnant women: A meta-analysis. Pediatrics.

[CR26] Mark KS, Farquhar B, Chisolm MS, Coleman-Cowger VH, Terplan M (2015). Knowledge, attitudes, and practice of electronic cigarette use among pregnant women. Journal of Addiction Medicine.

[CR27] McCubbin A, Fallin-Bennett A, Barnett J, Ashford K (2017). Perceptions and use of electronic cigarettes in pregnancy. Health Education Research.

[CR28] McGrath-Morrow SA, Hayashi M, Aherrera A, Lopez A, Malinina A, Collaco JM, Neptune E (2015). The effects of electronic cigarette emissions on systemic cotinine levels, weight and postnatal lung growth in neonatal mice. PLoS ONE.

[CR29] Murin S, Rafii R, Bilello K (2011). Smoking and smoking cessation in pregnancy. Clinics in Chest Medicine.

[CR30] Rebhan B, Kohlhuber M, Schwegler U, Koletzko B, Fromme H (2009). Smoking, alcohol and caffeine consumption of mothers before, during and after pregnancy - results of the study 'Breast-Feeding Habits in Bavaria'. Gesundheitswesen.

[CR31] Schenk L, Bau A-M (2006). Mindestindikatorensatz zur Erfassung des Migrationsstatus - Empfehlungen für die epidemiologische Praxis. [A basic set of indicators for mapping migrant status. Recommendations for epidemiological practice]. Bundesgesundheitsblatt Gesundheitsforschung Gesundheitschutz.

[CR32] Schilling L, Schneider S, Maul H, Spallek J (2020). STudy on E-cigarettes and Pregnancy (STEP)—Study protocol of a mixed-methods study on risk perception of e-cigarette use during pregnancy and sample description. Geburtshilfe und Frauenheilkunde.

[CR33] Schneider S, Schütz J (2008). Who smokes during pregnancy? A systematic literature review of population-based surveys conducted in developed countries between 1997 and 2006. The European Journal of Contraception & Reproductive Health Care.

[CR34] Spallek J, Grosser A, Holler-Holtrichter C, Doyle IM, Breckenkamp J, Razum O (2017). Early childhood health in Bielefeld, Germany (BaBi study): Study protocol of a social-epidemiological birth cohort. British Medical Journal Open.

[CR35] SPATZ Studie (2018). Die Säulen. Retrieved January 20, 2018, from https://www.ulmer-forschen.de/die-saeulen/10-ulmer-spatz-gesundheitsstudie/die-studie.

[CR36] Statistisches Amt für Hamburg und Schleswig-Holstein (2019). Statistisches Jahrbuch Hamburg [Statistical Yearbook Hamburg].

[CR37] Statistisches Bundesamt. (2018). *Bildungsstand der Bevölkerung. Ergebnisse des Mikrozensus 2017. [Educational level of the population. Results of the microcensus 2017]*. Wiesbaden: Statistisches Bundesamt.

[CR38] U.S. Department of Health and Human Services. (2014). *The health consequences of smoking - 50 years of progress: A report of the surgeon general*. Atlanta, GA: U.S. Department of Health and Human Services, Centers for Disease Control and Prevention, National Center for Chronic Disease Prevention and Health Promotion, Office on Smoking and Health.

[CR39] Wagner NJ, Camerota M, Propper C (2017). Prevalence and perceptions of electronic cigarette use during pregnancy. Maternal and Child Health Journal.

[CR40] Zeiher J, Kuntz B, Lange C (2017). Rauchen bei Erwachsenen in Deutschland [Smoking among adults in Germany]. Journal of Health Monitoring.

[CR41] Zeiher J, Starker A, Lampert T, Kuntz B (2018). Passivrauchbelastung bei Erwachsenen in Deutschland [Passive smoking among adults in Germany]. Journal of Health Monitoring.

